# Functional Brain Imaging in Knee Osteoarthritis: A Bibliometric Analysis of Research Trends and Emerging Clinical Perspectives

**DOI:** 10.7759/cureus.116453

**Published:** 2026-09-17

**Authors:** Jing Wang, Peng Xia, Qi Yang, Hao Wu, Zheng Wang, Xin-Jian Yu, Yong-Jing Xin, Jian-Hua Tang

**Affiliations:** 1 Department of Orthopedics, Wuxi Xishan District Hospital of Traditional Chinese Medicine, Wuxi, CHN; 2 Department of Radiology, Wuxi Xishan District Hospital of Traditional Chinese Medicine, Wuxi, CHN; 3 Department of Orthopedics, Fangta Hospital of Traditional Chinese Medicine, Shanghai, CHN; 4 Department of Tuina (Therapeutic Massage), Wuxi Hospital of Traditional Chinese Medicine, Wuxi, CHN

**Keywords:** bibliometric analysis, chronic pain management, functional magnetic resonance imaging, knee osteoarthritis (koa), knee osteoarthritis/ koa, resting-state functional connectivity, visual analysis

## Abstract

Knee osteoarthritis (KOA) is a leading cause of chronic disability, and the well-documented discordance between structural joint damage and pain experience suggests that central nervous system mechanisms contribute to symptoms independently of peripheral pathology. Functional magnetic resonance imaging (fMRI) has increasingly been used to investigate brain network alterations in KOA, yet the research landscape of this emerging field remains unmapped. We characterized publication trends, collaboration patterns, and methodological characteristics of brain fMRI research in KOA. Following PRISMA 2020 principles adapted for evidence mapping, we searched the Web of Science Core Collection on September 6, 2026, and included 43 English-language original studies (2008-2027) after 2-stage independent screening with chance-adjusted inter-reviewer agreement. Publication output, collaboration networks, keyword co-occurrence and burst detection, and paradigm-metric-target coupling were analyzed using VOSviewer, CiteSpace, and the bibliometrix package in R. Annual output fluctuated between one and six publications, peaking at six in 2022. The United States (n = 20) and China (n = 17) were the most productive countries and formed the leading bilateral collaboration dyad. Resting-state fMRI was the most frequently used paradigm (24 studies, 55.8%) and functional connectivity the most frequently applied analytical metric (25 studies, 58.1%); the medial prefrontal cortex (13 studies, 30.2%) and the sensorimotor cortex (12 studies, 27.9%) were the most frequently investigated brain targets. Author-keyword and Keywords Plus analyses yielded complementary thematic structures comprising ten modularity-based clusters (modularity Q = 0.6268, weighted mean silhouette = 0.8522); the most recent keyword bursts (2024 onward) were "pain," "functional connectivity," and the Keywords Plus-derived term "hip." These findings describe publication activity and methodological preferences within the WoSCC-indexed, English-language literature and do not establish the clinical validity of any imaging measure.

## Introduction and background

Knee osteoarthritis (KOA) represents a leading cause of chronic disability among older adults [1]. Clinical management is challenged by marked heterogeneity in pain experience across the disease spectrum: a substantial minority of patients report persistent pain following technically successful total knee arthroplasty (TKA), while a considerable proportion of patients with severe radiographic joint degeneration remain minimally symptomatic [2-5]. This well-documented structure-symptom discordance poses a fundamental challenge to orthopedic decision-making and suggests that central nervous system factors may substantially influence pain experience independent of peripheral joint damage [6-7].

Central sensitization, i.e., maladaptive plasticity within pain-processing brain circuits that amplifies and perpetuates pain, is increasingly implicated in KOA pathophysiology [8-11]; it can be quantified with validated instruments [12] and has been linked to persistent pain after technically successful TKA [13,14]. At the neuroimaging level, functional magnetic resonance imaging (fMRI) studies have identified alterations in resting-state functional connectivity within the default mode network, sensorimotor network, and limbic-related circuits among patients with chronic knee pain [15-17], findings contextualized by a meta-analysis confirming disrupted default mode connectivity and altered top-down pain modulation across chronic musculoskeletal pain conditions [18]. Despite these advances, the research landscape of this emerging field, encompassing publication dynamics, collaboration patterns, and methodological characteristics, remains incompletely characterized.

While previous bibliometric analyses have examined osteoarthritis-related research domains more broadly [19-21], including a recent panoramic mapping of knee MRI research dominated by structural and local imaging modalities [22], no study has specifically mapped functional neuroimaging research targeting central pain mechanisms in KOA. In this study, we analyzed 43 original brain fMRI studies in KOA (2008-2027) to: (i) characterize publication trends; (ii) map international and institutional collaboration networks; and (iii) describe methodological characteristics, including imaging paradigms, analytical metrics, brain network targets, and thematic structure. Because this analysis examines publication metadata rather than clinical outcomes, our goal is to provide a descriptive map of what has been published and how; it does not evaluate the clinical validity of any imaging measure.

## Review

Methods

Data Source and Search Strategy

An updated systematic literature search of the Web of Science Core Collection (WoSCC) was conducted on September 6, 2026, after complete indexing of the 2025 publication year. The search was restricted to the Science Citation Index Expanded (SCI-EXPANDED) and the Social Sciences Citation Index (SSCI), which comprehensively cover the biomedical, clinical neuroscience, and pain research journals relevant to this analysis; other Core Collection indexes were excluded because they do not systematically index the clinical neuroimaging and orthopedic surgery literature pertinent to this research question. The Boolean search formula was: TS = ("Osteoarthritis, Knee" OR "Knee Osteoarthritis" OR "Osteoarthritis of the Knee" OR "Knee OA") AND TS = ("fMRI" OR "functional MRI" OR "functional magnetic resonance imaging" OR "functional MRIs" OR "resting state fMRI" OR "task fMRI" OR "rs-fMRI"). To maximize sensitivity, no document-type or language filters were applied at the database level; publication type and language were assessed during screening.

Bibliographic records were exported in plain-text format with full records and cited references, including author, title, source, abstract, author keywords, Keywords Plus, addresses (institution and country), citation counts, and reference lists. The screening and reporting process was informed by the PRISMA 2020 reporting principles, adapted for bibliometric analysis [23-24]. The search window was not artificially truncated: it spans the first indexed publication on this topic (2008) through the search date. One record published online in 2026 was assigned to a 2027 volume by the indexer, and the 2026 count represents a partial year; annual output is therefore reported as absolute counts only.

Study Selection

Inclusion criteria were: (i) English-language original research articles; (ii) reporting brain functional MRI (task-based and/or resting-state) in human knee osteoarthritis populations, with KOA as the dominant patient population or the explicitly stated primary disease target of the neuroimaging analysis; and (iii) reporting original neuroimaging data in which fMRI constituted an outcome measure. Exclusion criteria were: (i) animal studies; (ii) reviews and meta-analyses; (iii) study protocols and conference abstracts without original data; (iv) brain imaging studies not focused on KOA; (v) studies in which fMRI was not used as an outcome measure, including structural-only brain imaging studies; and (vi) retracted publications.

Records underwent a two-stage screening process (title/abstract and full-text review) performed independently by two reviewers (J.W. and P.X.), with discrepancies resolved by consensus discussion. From 101 records identified, 2 non-English publications were excluded and 99 records underwent screening; 56 records were excluded (animal studies, n = 8; publications without original data, n = 22, comprising 12 study protocols and 10 conference abstracts; brain imaging studies not focused on KOA, n = 12; reviews, n = 12; retracted article, n = 1; fMRI not used as an outcome measure, n = 1), leaving 43 studies in the final dataset (Figure 1). A complete list of the 43 included studies (first author, year, title, journal, DOI) is provided in Table 1. Forty-three studies were included in the final dataset (Figure 1 and Table 1) [25-65].

**Figure 1:**
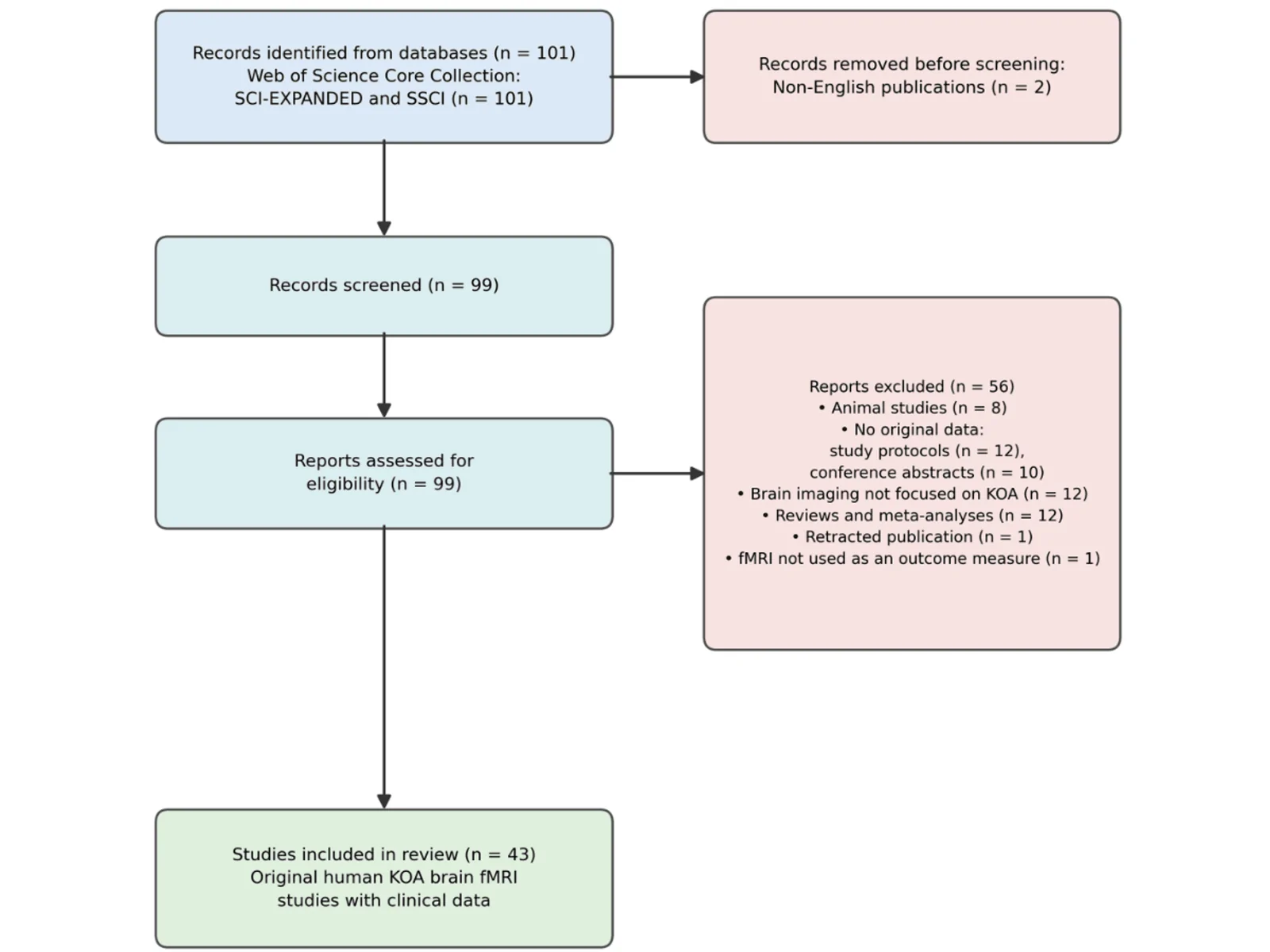
PRISMA 2020 flow diagram of study identification and selection; WoSCC (Web of Science Core Collection)

**Table 1:** Per-study extraction data of the 43 included studies Coding rules Imaging paradigm: rs-fMRI = resting-state fMRI (including ASL resting perfusion imaging); task-fMRI = task-based or stimulus-evoked fMRI; Mixed = resting-state and task-based fMRI in the same cohort, or fMRI combined with another modality (structural MRI/VBM, cortical thickness, DTI, or PET); structural MRI was always coded under Mixed. Analytical metrics: each metric was counted once per study, and one study could contribute multiple metrics. ALFF and fALFF were merged for all statistical summaries and are reported jointly as ALFF/fALFF. Brain network targets: Each target was counted once per study. Category merges: ACC/MCC includes rACC, sgACC, and aMCC; Sensorimotor includes S1, S2, M1, SMA, and the precentral gyrus; mPFC includes the medial frontal pole and medial superior frontal gyrus; DLPFC includes the middle frontal gyrus. Targets reported only at the lobe level without subregion specification are marked "(unspec)" and were not included in the top-10 target rankings (image 6B). Study 38 reported whole-brain circuitry without a specific target. Studies 30 (2008) and 43 (2014) are multi-condition cohorts containing independently analyzed knee osteoarthritis subgroups. FPN: frontoparietal network; ECN: executive control network; VBM: voxel-based morphometry; GMV: gray matter volume; GMD: gray matter density; ALFF: amplitude of low-frequency fluctuations; ACC: anterior cingulate cortex; mPFC: medial prefrontal cortex; OFC: orbitofrontal cortex; DMN: default mode network; DLPFC: dorsolateral prefrontal cortex; PAG: periaqueductal gray; DTI: diffusion tensor imaging; ASL: arterial spin labeling; FC: flow cytometry

No.	Ref.	Year	Title	Journal	DOI	Imaging paradigm	Analytical metrics	Brain network targets
1	25	2026	Explainable Graph Attention Network on Resting State fMRI Dynamic Functional Connectivity Reveals Cerebellar Hub Breakdown in Chronic Knee Osteoarthritis Pain	Iranian Journal Of Science And Technology-Transactions Of Electrical Engineering	10.1007/s40998-025-01012-z	rs-fMRI	Functional connectivity (FC/rs-FC); Dynamic FC (dFC); Graph theory; Machine learning (ML)	Cerebellum
2	26	2021	Structural and Functional Abnormalities in Knee Osteoarthritis Pain Revealed With Multimodal Magnetic Resonance Imaging	Frontiers In Human Neuroscience	10.3389/fnhum.2021.783355	Mixed (incl. structural MRI)	Structural morphometry (VBM/GMV/GMD); Fractional ALFF (fALFF)	Insula; Hippocampus; Cerebellum; Sensorimotor; Occipital/Visual
3	27	2015	The modulation effect of longitudinal acupuncture on resting state functional connectivity in knee osteoarthritis patients	Molecular Pain	10.1186/s12990-015-0071-9	Mixed (incl. structural MRI)	Independent component analysis (ICA); Functional connectivity (FC/rs-FC)	FPN/ECN; ACC; mPFC; Insula
4	28	2026	Neuroregulatory mechanism of heat-sensitive moxibustion on the Dubi acupoint (ST 35) in patients with knee osteoarthritis: a resting-state functional magnetic resonance imaging study	Frontiers In Neurology	10.3389/fneur.2026.1699988	rs-fMRI	Fractional ALFF (fALFF)	Frontal(unspec); Temporal(unspec); Occipital/Visual
5	29	2025	Effect of Acupuncture on Functional Connectivity of the Limbic Network in Patients with Knee Osteoarthritis: A Resting State Functional Magnetic Resonance Imaging Study	Journal Of Pain Research	10.2147/JPR.S519308	rs-fMRI	Independent component analysis (ICA); Functional connectivity (FC/rs-FC)	Limbic network; Temporal pole; Hippocampus; ACC; Amygdala; OFC
6	30	2020	Altered Intrinsic Brain Activity and Functional Connectivity Before and After Knee Arthroplasty in the Elderly: A Resting-State fMRI Study	Frontiers In Neurology	10.3389/fneur.2020.556028	rs-fMRI	ALFF; Functional connectivity (FC/rs-FC)	DMN; Precuneus; Amygdala; Cerebellum
7	17	2024	Altered static and dynamic functional brain network in knee osteoarthritis: A resting-state functional magnetic resonance imaging study. Static and dynamic FNC in KOA	Neuroimage	10.1016/j.neuroimage.2024.120599	rs-fMRI	Independent component analysis (ICA); Functional connectivity (FC/rs-FC); Dynamic FC (dFC)	DMN; Occipital/Visual; Cerebellum; Subcortical network
8	31	2022	Altered brain activity in end-stage knee osteoarthritis revealed by resting-state functional magnetic resonance imaging	Brain And Behavior	10.1002/brb3.2479	Mixed (incl. structural MRI)	Structural morphometry (VBM/GMV/GMD); Functional connectivity (FC/rs-FC)	Temporal (unspec); DLPFC; mPFC
9	32	2018	A Functional Neuroimaging Study of Expectancy Effects on Pain Response in Patients With Knee Osteoarthritis	Journal Of Pain	10.1016/j.jpain.2017.12.260	task-fMRI	Task-evoked activation (task BOLD); Regional homogeneity (ReHo)	DLPFC; Pain matrix
10	33	2025	Inflammation-mediated regional brain alterations associated with mild cognitive impairment in knee osteoarthritis	Arthritis Research & Therapy	10.1186/s13075-025-03646-0	rs-fMRI	Regional homogeneity (ReHo); ALFF; Machine learning (ML)	mPFC; Precuneus; Temporal(unspec)
11	34	2014	The dorsolateral prefrontal network is involved in pain perception in knee osteoarthritis patients	Neuroscience Letters	10.1016/j.neulet.2014.08.027	task-fMRI	Task-evoked activation (task BOLD); Effective connectivity (PPI/EC)	DLPFC; Pain matrix
12	35	2025	Effect of acupuncture treatment for patients with knee osteoarthritis on brain fluctuation amplitude and functional connectivity: a randomized three-armed fMRI study	BMC Complementary Medicine And Therapies	10.1186/s12906-025-04985-w	rs-fMRI	ALFF; Functional connectivity (FC/rs-FC)	DLPFC; Thalamus; Cerebellum
13	36	2020	Altered Resting-State Connectivity with Pain-Related Expectation Regions in Female Patients with Severe Knee Osteoarthritis	Journal Of Pain Research	10.2147/JPR.S268529	rs-fMRI	Functional connectivity (FC/rs-FC)	Insula; OFC
14	37	2022	Acupuncture Enhances Dorsal Raphe Functional Connectivity in Knee Osteoarthritis With Chronic Pain	Frontiers In Neurology	10.3389/fneur.2021.813723	rs-fMRI	Functional connectivity (FC/rs-FC)	PAG; Raphe nuclei; Occipital/Visual
15	15	2022	Resting-state functional connectivity alteration in elderly patients with knee osteoarthritis and declined cognition: An observational study	Frontiers In Aging Neuroscience	10.3389/fnagi.2022.1002642	rs-fMRI	Graph theory; Functional connectivity (FC/rs-FC)	DMN
16	38	2017	Brain imaging of pain sensitization in patients with knee osteoarthritis	Pain	10.1097/j.pain.0000000000000985	task-fMRI	Task-evoked activation (task BOLD)	Pain matrix; Sensorimotor; Occipital/Visual
17	39	2018	Correlation of Pain Reduction with fMRI BOLD Response in Osteoarthritis Patients Treated with Paracetamol: Randomized, Double-Blind, Crossover Clinical Efficacy Study	Pain Medicine	10.1093/pm/pnx157	task-fMRI	Task-evoked activation (task BOLD)	Sensorimotor; Parietal(unspec)
18	40	2023	Modulation effects of different treatments on periaqueductal gray resting state functional connectivity in knee osteoarthritis knee pain patients	CNS Neuroscience & Therapeutics	10.1111/cns.14153	rs-fMRI	Functional connectivity (FC/rs-FC)	PAG
19	41	2014	Acupuncture modulates cortical thickness and functional connectivity in knee osteoarthritis patients	Scientific Reports	10.1038/srep06482	Mixed (incl. structural MRI)	Cortical thickness (CT); Functional connectivity (FC/rs-FC)	mPFC; ACC; PAG
20	42	2024	Changed resting-state connectivity of anterior insular cortex affects subjective pain reduction after knee arthroplasty: A longitudinal study	Brain Research Bulletin	10.1016/j.brainresbull.2024.111073	rs-fMRI	Functional connectivity (FC/rs-FC)	Insula; OFC; mPFC
21	43	2013	Image formation of brain function in patients suffering from knee osteoarthritis treated with moxibustion	Journal Of Traditional Chinese Medicine	10.1016/S0254-6272(13)60122-3	rs-fMRI	Fractional ALFF (fALFF); Regional homogeneity (ReHo)	Cerebellum; Thalamus; Sensorimotor; Frontal(unspec); Occipital/Visual; Parietal(unspec)
22	44	2011	Brain activity for chronic knee osteoarthritis: Dissociating evoked pain from spontaneous pain	European Journal Of Pain	10.1016/j.ejpain.2010.12.007	task-fMRI	Task-evoked activation (task BOLD)	PFC(unspec); Limbic network
23	45	2024	The modulation effects of the mind-body and physical exercises on the basolateral amygdala-temporal pole pathway on individuals with knee osteoarthritis	International Journal Of Clinical And Health Psychology	10.1016/j.ijchp.2023.100421	rs-fMRI	Functional connectivity (FC/rs-FC)	Amygdala; Temporal pole; mPFC
24	46	2022	Tai Chi Improves Brain Functional Connectivity and Plasma Lysophosphatidylcholines in Postmenopausal Women With Knee Osteoarthritis: An Exploratory Pilot Study	Frontiers In Medicine	10.3389/fmed.2021.775344	Mixed (incl. structural MRI)	Functional connectivity (FC/rs-FC); DTI	Amygdala; mPFC
25	47	2015	Organisation of the motor cortex differs between people with and without knee osteoarthritis	Arthritis Research & Therapy	10.1186/s13075-015-0676-4	task-fMRI	Task-evoked activation (task BOLD)	Sensorimotor
26	48	2026	Brain functional basis of the association between positive expectation and acupuncture efficacy in knee osteoarthritis	Brain Imaging And Behavior	10.1007/s11682-026-01094-1	rs-fMRI	Fractional ALFF (fALFF); Functional connectivity (FC/rs-FC)	Precuneus; mPFC
27	49	2024	Thalamocortical dysconnectivity is associated with pain in patients with knee osteoarthritis	European Journal Of Neuroscience	10.1111/ejn.16531	rs-fMRI	Functional connectivity (FC/rs-FC); Effective connectivity (PPI/EC); Graph theory	Thalamus; Sensorimotor; Frontal(unspec); Parietal(unspec); Temporal(unspec); Occipital/Visual
28	50	2019	Different exercise modalities relieve pain syndrome in patients with knee osteoarthritis and modulate the dorsolateral prefrontal cortex: A multiple mode MRI study	Brain Behavior And Immunity	10.1016/j.bbi.2019.08.193	Mixed (incl. structural MRI)	Functional connectivity (FC/rs-FC); Structural morphometry (VBM/GMV/GMD)	DLPFC; Sensorimotor
29	51	2022	Tapentadol effects on brain response to pain in sensitized patients with knee osteoarthritis	Rheumatology	10.1093/rheumatology/keab761	task-fMRI	Task-evoked activation (task BOLD)	PFC(unspec); Sensorimotor
30	52	2008	A preliminary fMRI study of analgesic treatment in chronic back pain and knee osteoarthritis	Molecular Pain	10.1186/1744-8069-4-47	task-fMRI	Task-evoked activation (task BOLD)	Thalamus; Sensorimotor; Insula; ACC; Putamen; Amygdala
31	53	2027	Neuroinflammation in knee osteoarthritis is accompanied by functional reorganization of the S1 cortical knee representation	Brain Behavior And Immunity	10.1016/j.bbi.2026.106972	Mixed (incl. structural MRI)	Task-evoked activation (task BOLD); PET	Sensorimotor
32	54	2014	Naproxen Effects on Brain Response to Painful Pressure Stimulation in Patients with Knee Osteoarthritis: A Double-blind, Randomized, Placebo-controlled, Single-dose Study	Journal Of Rheumatology	10.3899/jrheum.131367	task-fMRI	Task-evoked activation (task BOLD)	Amygdala; Pain matrix
33	55	2018	Enhancing treatment of osteoarthritis knee pain by boosting expectancy: A functional neuroimaging study	Neuroimage-Clinical	10.1016/j.nicl.2018.01.021	rs-fMRI	Functional connectivity (FC/rs-FC)	NAc; mPFC; ACC; DLPFC
34	56	2026	Effects of Tai Chi combined with transcranial direct current stimulation on pain in knee osteoarthritis: a randomized controlled neuroimaging trial	Bmc Medicine	10.1186/s12916-026-04760-9	rs-fMRI	Functional connectivity (FC/rs-FC)	DLPFC; Pain matrix
35	57	2023	Tai Chi exercise reduces circulating levels of inflammatory oxylipins in postmenopausal women with knee osteoarthritis: results from a pilot study	Frontiers In Medicine	10.3389/fmed.2023.1210170	rs-fMRI	Functional connectivity (FC/rs-FC)	Amygdala; mPFC
36	58	2019	Central Sensitization in Knee Osteoarthritis: Relating Presurgical Brainstem Neuroimaging and PainDETECT-Based Patient Stratification to Arthroplasty Outcome	Arthritis & Rheumatology	10.1002/art.40749	task-fMRI	Task-evoked activation (task BOLD); Functional connectivity (FC/rs-FC)	ACC; Brainstem
37	59	2016	Associations of limbic-affective brain activity and severity of ongoing chronic arthritis pain are explained by trait anxiety	Neuroimage-Clinical	10.1016/j.nicl.2016.06.022	rs-fMRI	ASL perfusion (ASL/CBF)	ACC; Hippocampus; Amygdala; Insula; Putamen; Brainstem
38	60	2018	Inferring distinct mechanisms in the absence of subjective differences: Placebo and centrally acting analgesic underlie unique brain adaptations	Human Brain Mapping	10.1002/hbm.23999	Mixed (incl. structural MRI)	Structural morphometry (VBM/GMV/GMD); Graph theory	Whole-brain circuitry (no specific target)
39	61	2015	Repeated verum but not placebo acupuncture normalizes connectivity in brain regions dysregulated in chronic pain	Neuroimage-Clinical	10.1016/j.nicl.2015.09.012	rs-fMRI	Functional connectivity (FC/rs-FC)	PAG; mPFC; Hippocampus
40	62	2016	Brain Connectivity Predicts Placebo Response across Chronic Pain Clinical Trials	PLoS Biology	10.1371/journal.pbio.1002570	rs-fMRI	Functional connectivity (FC/rs-FC)	DLPFC
41	63	2022	Distinctive alterations in the functional anatomy of the cerebral cortex in pain-sensitized osteoarthritis and fibromyalgia patients	Arthritis Research & Therapy	10.1186/s13075-022-02942-3	rs-fMRI	Local FC	Insula; Sensorimotor
42	64	2018	Determining Brain Mechanisms that Underpin Analgesia Induced by the Use of Pain Coping Skills	Pain Medicine	10.1093/pm/pnx301	task-fMRI	Task-evoked activation (task BOLD)	mPFC; ACC; Sensorimotor; Insula; OFC; Parietal(unspec)
43	65	2014	Functional Reorganization of the Default Mode Network across Chronic Pain Conditions	PLoS One	10.1371/journal.pone.0106133	rs-fMRI	Functional connectivity (FC/rs-FC)	DMN; mPFC; Insula; Parietal(unspec)

Coding Scheme and Inter-Reviewer Agreement

The classification framework was developed based on commonly reported fMRI methodological components and prior neuroimaging literature. Two reviewers (J.W. and P.X.) independently classified each of the 43 included publications according to predefined categories; discrepancies were resolved by consensus discussion. Imaging paradigms were categorized as: (i) task-based fMRI (active cognitive, motor, or nociceptive tasks); (ii) resting-state fMRI (resting state with no explicit task); and (iii) mixed designs, defined as studies combining resting-state and task-based fMRI within the same cohort, or fMRI combined with an additional imaging modality (such as voxel-based morphometry, cortical thickness, diffusion tensor imaging, or positron emission tomography). Structural MRI was not classified as an fMRI paradigm per se; because structural MRI integration can co-occur with any paradigm, mixed-design studies are reported separately from paradigm proportions.

Analytical metrics included functional connectivity, task-evoked activation, amplitude of low-frequency fluctuation (ALFF/fALFF), regional homogeneity (ReHo), graph theory, independent component analysis (ICA), dynamic functional connectivity, effective connectivity (psychophysiological interaction), machine learning, and structural morphometry. Brain network targets were coded as the medial prefrontal cortex, sensorimotor cortex, insula, anterior/mid-cingulate cortex, dorsolateral prefrontal cortex, amygdala, cerebellum, pain matrix, default mode network, and periaqueductal gray. Coding was multi-label: a study could be assigned to multiple metric and target categories, so category counts are non-mutually exclusive and totals exceed 43.

Chance-adjusted inter-reviewer agreement was quantified using Cohen's kappa for each coding domain: paradigm classification (κ = 0.92), analytical metrics (mean per-category κ = 0.97, range 0.66-1.00), and brain network targets (mean per-category κ = 0.89, range 0.56-1.00). For the multi-label domains (analytical metrics and brain network targets), kappa was computed on the binary presence/absence coding of each category and summarized as the mean and range. All discrepancies were resolved by consensus discussion before analysis.

A three-domain Sankey diagram was constructed to visualize co-occurrence relationships among imaging paradigms, analytical metrics, and brain network targets. Flow width reflects the number of studies reporting a given combination; because individual studies could report multiple metrics and multiple targets, total flow counts exceed the number of included studies (n = 43). The stacked bar chart and heatmaps) were generated in Python (Matplotlib, version 3.7) from the manually coded dataset, and the Sankey diagram) was generated using Plotly (version 5.15) based on co-occurrence matrices exported from the coding spreadsheet.

Bibliometric Analysis

All analyses were performed on the September 6, 2026 dataset. Three tools were employed to enable cross-validation of network structures and topic trends; detailed software parameter configurations are summarized in Table 2. Descriptive analysis was performed using the bibliometrix package (version 4.1.0) in R (version 4.3.0) [66]. Because annual publication counts in this emerging field are small (range: 1-6), absolute frequencies are reported as the primary metric and no annual growth rates are calculated, as percentages derived from small denominators and a partial-year 2026 count would be mathematically unstable.

**Table 2:** Software tools and parameter configurations used in the bibliometric analysis

Tool / Software	Version	Developer / Source	Function in This Study	Key Parameter Settings
R with bibliometrix package	4.1.0 (R 4.3.0)	R Foundation for Statistical Computing	Descriptive bibliometric analysis: annual output and absolute frequencies	Absolute counts only; no growth-rate calculation
VOSviewer	1.6.20	Leiden University	Keyword co-occurrence networks; author keywords and Keywords Plus analyzed as separate fields	Full counting; association strength normalization; clustering resolution gamma = 1.0; thesaurus file for synonym harmonization
CiteSpace	6.2.R4	Drexel University	Country and institutional collaboration networks; keyword burst detection; modularity-based clustering	g-index (k = 25), LRF = 2.5, L/N = 10, LBY = 5, e = 1.0; pathfinder pruning; density, modularity Q, and silhouette reported
Python (Matplotlib / Plotly)	3.7 / 5.15	PSF / Plotly Technologies	Stacked bar chart and heatmaps; Sankey diagram	Co-occurrence matrices exported from the coding spreadsheet

Country and institutional collaboration networks, keyword burst detection, and modularity-based clustering were analyzed using CiteSpace (version 6.2.R4, Drexel University) [67]. Selection criteria were: g-index (k = 25), LRF = 2.5, L/N = 10, LBY = 5, and e = 1.0, with pathfinder network pruning. Network density, modularity Q, and weighted mean silhouette scores are reported alongside the visualizations. Burst detection was applied to identify rapidly emerging keywords. Institution names were standardized using CiteSpace's built-in thesaurus function to merge variant abbreviations (e.g., "Capital Med Univ" and "Capital Medical University"), and country names were verified against the affiliation address field.

Keyword co-occurrence networks were constructed using VOSviewer (version 1.6.20, Leiden University) [68] with full counting and association strength normalization (clustering resolution gamma = 1.0). Author keywords and Keywords Plus were analyzed as separate fields. Keyword variants were harmonized prior to co-occurrence analysis using a VOSviewer thesaurus file (e.g., "functional mri," "fmri," and "functional magnetic resonance" were merged into a single term, and spelling variants were unified); the thesaurus file is provided as supplementary material. A sensitivity analysis comparing the co-occurrence networks before and after harmonization confirmed that the thematic structure was unchanged.

Methodological reporting followed established bibliometric standards [69] and neuroimaging analytical guidelines [70]. This study involved the analysis of publicly available published literature and did not include human participants, patient data, or animal subjects; therefore, ethical approval and informed consent were not required.

Results

Results are presented according to the predefined analytical framework described above. All analyses are based on the updated dataset of 43 original studies identified in the re-run search (search date: September 6, 2026).

Annual Publication Output

The 43 included studies span 2008 to 2027 (Figure 2). The first KOA fMRI publication appeared in 2008, and annual output remained sporadic through 2013 (no more than one publication per year, with none in 2009, 2010, and 2012). From 2014 onward, annual output fluctuated between one and six publications, peaking at six in 2022, with four, three, and four publications in 2024, 2025, and 2026, respectively. One further record was published online in 2026 and assigned to a 2027 volume. Because the search was closed on September 6, 2026, the 2026 count is partial, and the 2027 record reflects ahead-of-print indexing; neither represents complete-year output. Given the small annual counts (range 0-6), year-to-year fluctuations are reported as absolute numbers only and should be interpreted as descriptive observations rather than epidemiological trends. Cumulative output reached 43 studies by the search date.

**Figure 2:**
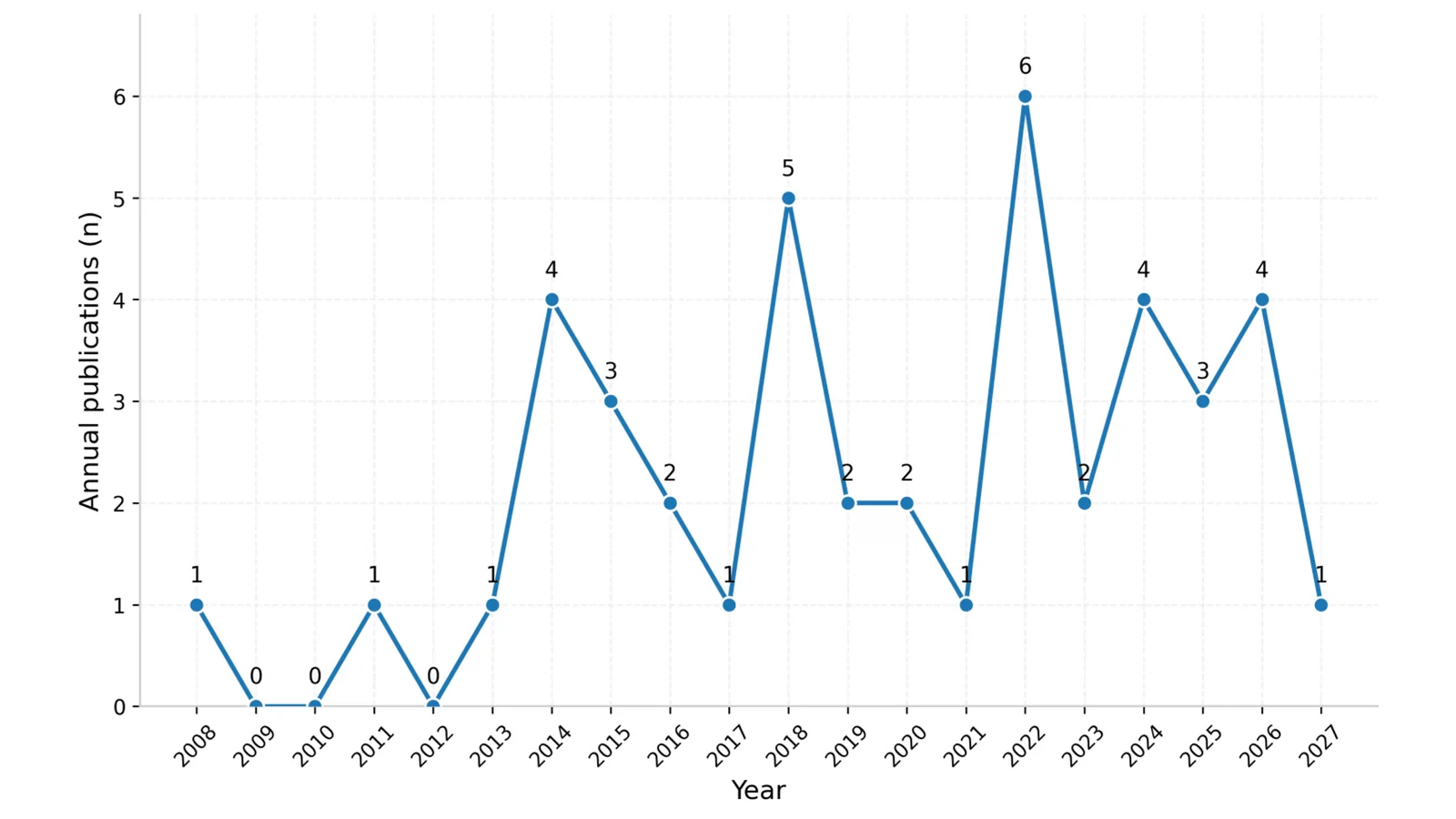
Annual publication output of brain fMRI research in KOA, 2008-2027 fMRI: functional magnetic resonance imaging; KOA: knee osteoarthritis

Collaboration Networks

The country collaboration network (Figure 3A) identified the United States (n = 20) and China (n = 17) as the most productive countries, forming the dominant bilateral partnership, with England and Spain (n = 5 each), Australia (n = 4), and Japan (n = 3) forming secondary collaborative nodes; Iran (n = 1) and the Netherlands (n = 1) appeared only in the most recent years. The institutional network (Figure 3B; 87 nodes, 227 links, density = 0.0607) comprised three regional groupings: a North American grouping led by Harvard University and Massachusetts General Hospital (n = 8 each), together with Harvard Medical School, Brigham and Women's Hospital, and Northwestern University (n = 5; the earliest contributing institution, 2008); a Spanish grouping centered on Hospital del Mar, the Hospital del Mar Research Institute, the Autonomous University of Barcelona, and the CIBER/CIBERSAM biomedical research networks (n = 4 each); and an Australian grouping centered on the University of Melbourne, Monash University, and the University of Queensland. Capital Medical University (n = 4) was the most productive Chinese institution. Industry-affiliated institutions (Pfizer, GlaxoSmithKline) appeared as peripheral collaborative nodes.

**Figure 3:**
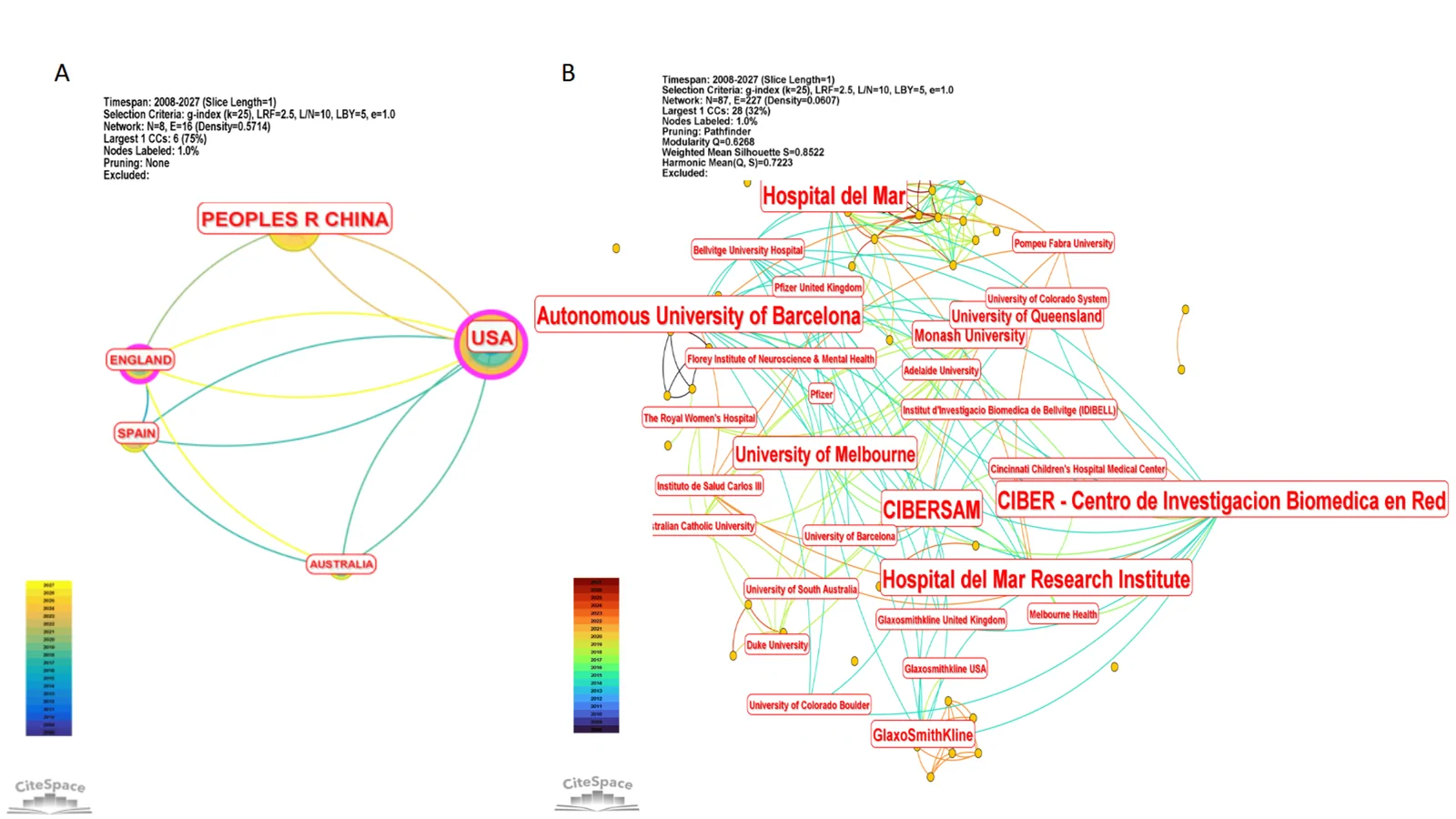
Collaboration networks in KOA brain fMRI research (A) Country/region collaboration network. (B) Institutional collaboration network. fMRI: functional magnetic resonance imaging; KOA: knee osteoarthritis

Keyword Co-occurrence, Burst Detection, and Thematic Clustering

Author keywords and Keywords Plus were analyzed separately. The author-keyword network (Figure 4A) was centered on "knee osteoarthritis," "chronic pain," "acupuncture," "functional magnetic resonance imaging," and "functional connectivity," reflecting author-designated topics dominated by disease, intervention, and imaging-modality terms. The Keywords Plus network (Figure 4B) was broader, with "fMRI," "brain," "cortex," "mechanisms," and "functional connectivity" as central nodes, reflecting the literature cited by the included studies; the term "hip" occurred exclusively in this field and not among author keywords, and its interpretation is addressed in the Discussion. Keyword burst detection (Figure 4C) identified 10 bursts: the earliest involved "resting state fMRI" (2015), "placebo analgesia" (2018), and "ratio scales" (2018); "cortex" burst during 2020-2022; "knee osteoarthritis" burst from 2022 to 2025, and "pain sensitization" and "functional mri" each burst in 2022; the most recent bursts were "pain" (strength 1.91, 2024-2027), "functional connectivity" (strength 1.40, 2024-2025), and "hip" (strength 1.09, 2024). Modularity-based clustering of the keyword network (density = 0.0357) yielded ten thematic clusters (Figure 4D): elderly patients (#0), brain activity (#1), insular cortex (#2), pharmacotherapy (#3), dorsolateral prefrontal cortex (#4), limbic (#5), naproxen (#6), pain (#7), independent component analysis (#8), and chronic knee osteoarthritis (#9), with modularity Q = 0.6268 and weighted mean silhouette = 0.8522, indicating a well-structured and highly credible cluster solution. Keyword synonyms were harmonized prior to co-occurrence analysis using a VOSviewer thesaurus file (e.g., "functional mri," "fmri," and "functional magnetic resonance" were merged into a single term, and spelling variants were unified); the thematic structure of the co-occurrence networks was unchanged after harmonization.

**Figure 4:**
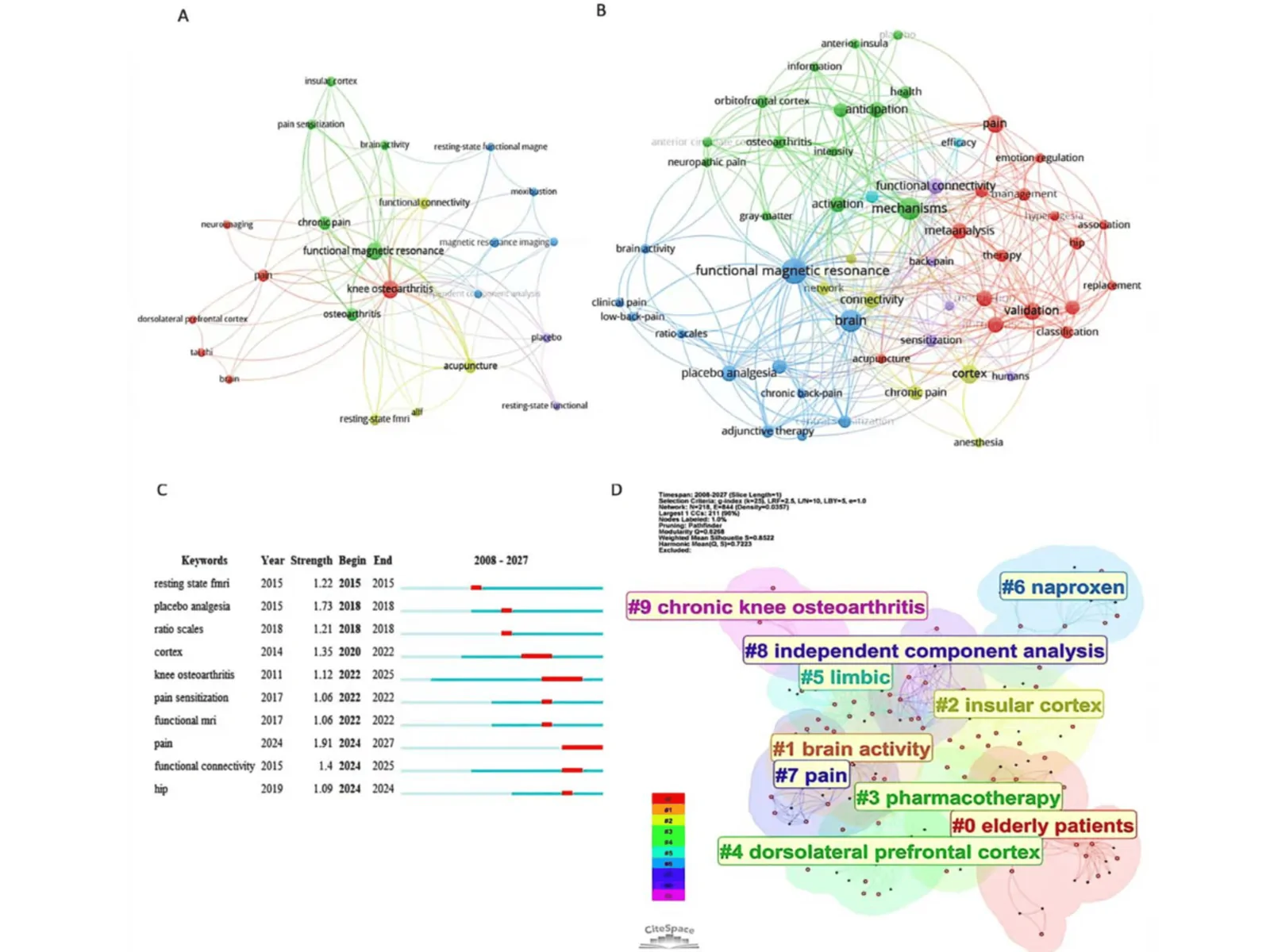
Keyword analyses of the 43 included studies (A) Author-keyword co-occurrence network. (B) Keywords Plus co-occurrence network. (C) Keyword burst detection. (D) Modularity-based keyword clustering.

Temporal Distribution of Imaging Paradigms

Across the 43 studies, resting-state fMRI (rs-fMRI) was the most frequently used paradigm (n = 24, 55.8%), followed by task-based fMRI (task-fMRI; n = 11, 25.6%) and mixed designs (n = 8, 18.6%). Mixed designs were defined as studies combining resting-state and task-based fMRI within the same cohort, or fMRI combined with an additional imaging modality; studies integrating structural MRI were coded within this category. The annual proportional distribution (Figure 5) shows that task-fMRI accounted for the earliest studies (2008-2011), the first resting-state study appeared in 2013, and the two paradigms coexisted during 2014-2019. From 2020 onward, resting-state fMRI predominated, accounting for 16 of the 19 studies (84.2%) published between 2022 and 2026; no study published after 2022 employed a task-only design. Structural MRI integration is reported separately from paradigm proportions because it can co-occur with any paradigm: all eight mixed-design studies incorporated structural MRI or another auxiliary modality (voxel-based morphometry, cortical thickness, diffusion tensor imaging, or positron emission tomography) alongside fMRI, appearing in 2014, 2015, 2018, 2019, 2021, 2022 (n = 2), and 2027. All percentages denote the proportion of studies within the stated period and, given the small denominators, are descriptive only.

**Figure 5:**
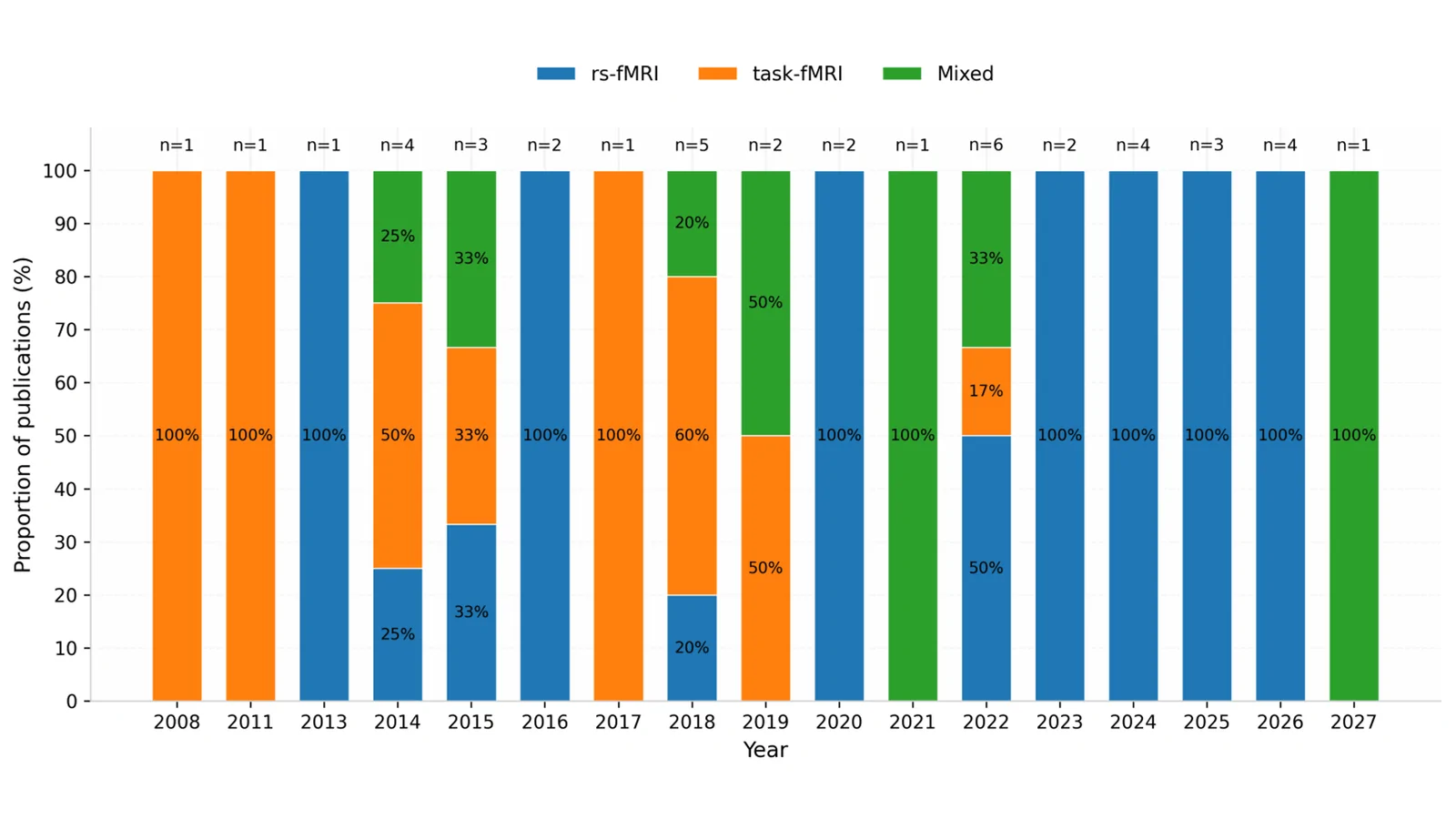
Annual proportional distribution of imaging paradigms, 2008-2027

Temporal Patterns of Analytical Metrics and Brain Network Targets

Functional connectivity was the most frequently applied analytical metric (25 of 43 studies, 58.1%), followed by task-evoked activation (12, 27.9%), amplitude of low-frequency fluctuation (ALFF/fALFF; 7, 16.3%), structural morphometry (4, 9.3%), graph theory (4, 9.3%), independent component analysis (3, 7.0%), and regional homogeneity (3, 7.0%); dynamic functional connectivity, effective connectivity, and machine learning were each reported in two studies (4.7%). Functional connectivity has been continuously represented since 2014, whereas machine learning and dynamic functional connectivity appeared only from 2024 onward (Figure 6A). Among brain network targets (Figure 6B), the medial prefrontal cortex (13 studies, 30.2%) and the sensorimotor cortex (12, 27.9%) were the most frequently investigated, followed by the insula (9, 20.9%), the anterior/mid-cingulate cortex (8, 18.6%), the dorsolateral prefrontal cortex (8, 18.6%), and the amygdala (8, 18.6%); the cerebellum (6, 14.0%), pain matrix (5, 11.6%), default mode network (4, 9.3%), and periaqueductal gray (4, 9.3%) were less frequently targeted. Because individual studies could report multiple metrics and multiple targets, counts are non-mutually exclusive and totals exceed 43.

**Figure 6:**
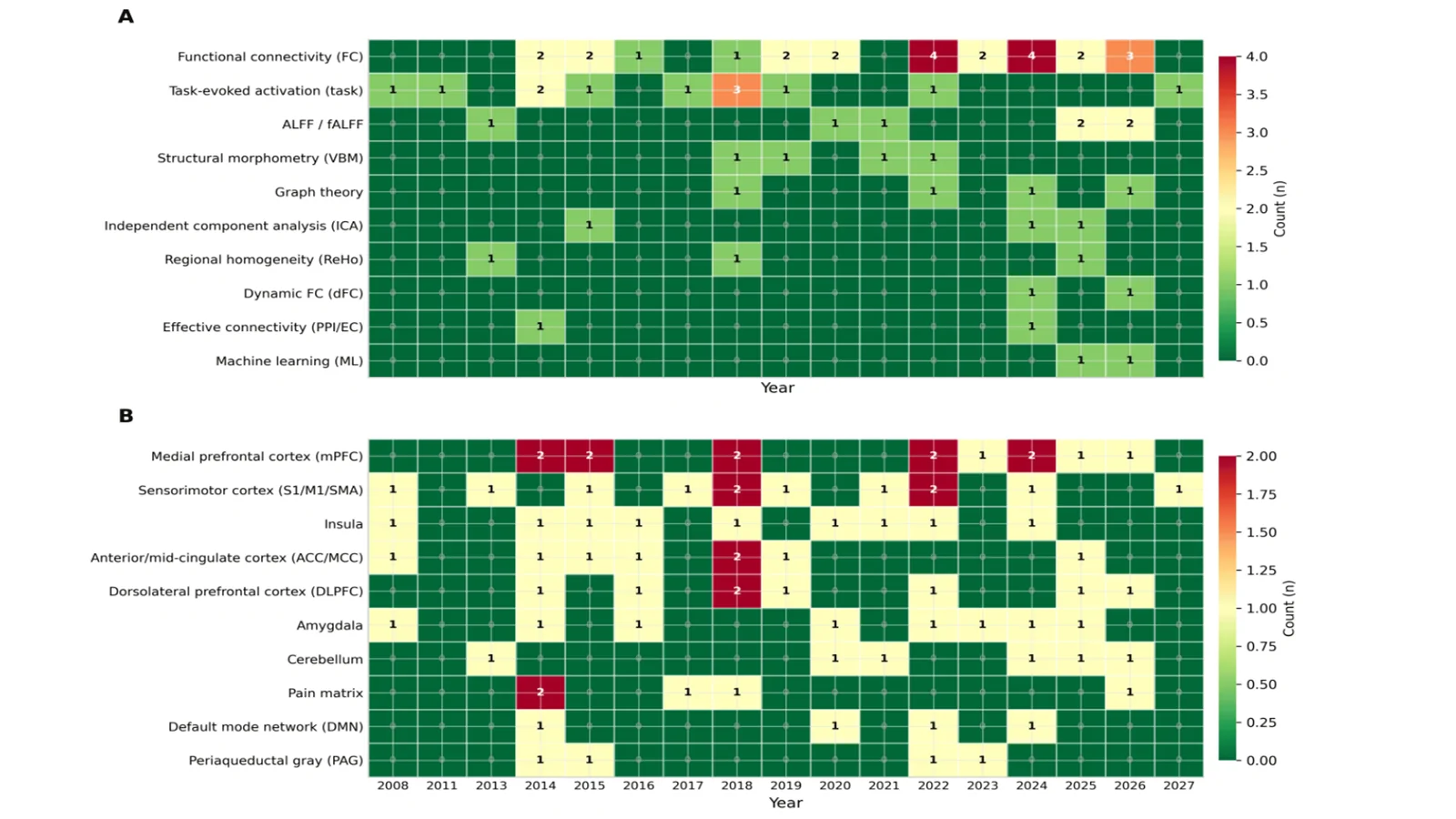
Year-by-year distribution of methodological characteristics (top 10 by frequency) (A) Analytical metrics. (B) Brain network targets.

Co-occurrence Patterns of Paradigms, Metrics, and Network Targets

The three-domain Sankey diagram (Figure 7) maps co-occurrence relationships among imaging paradigms, analytical metrics, and brain network targets. The dominant pathway ran from resting-state fMRI through functional connectivity to the medial prefrontal cortex and sensorimotor cortex. Task-based fMRI studies flowed predominantly through task-evoked activation toward the sensorimotor cortex, insula, and anterior cingulate cortex. Mixed-design studies bridged structural morphometry and connectivity-based metrics. Flow widths reflect non-mutually-exclusive co-occurrence frequencies; total flow counts therefore exceed the number of included studies (n = 43).

**Figure 7:**
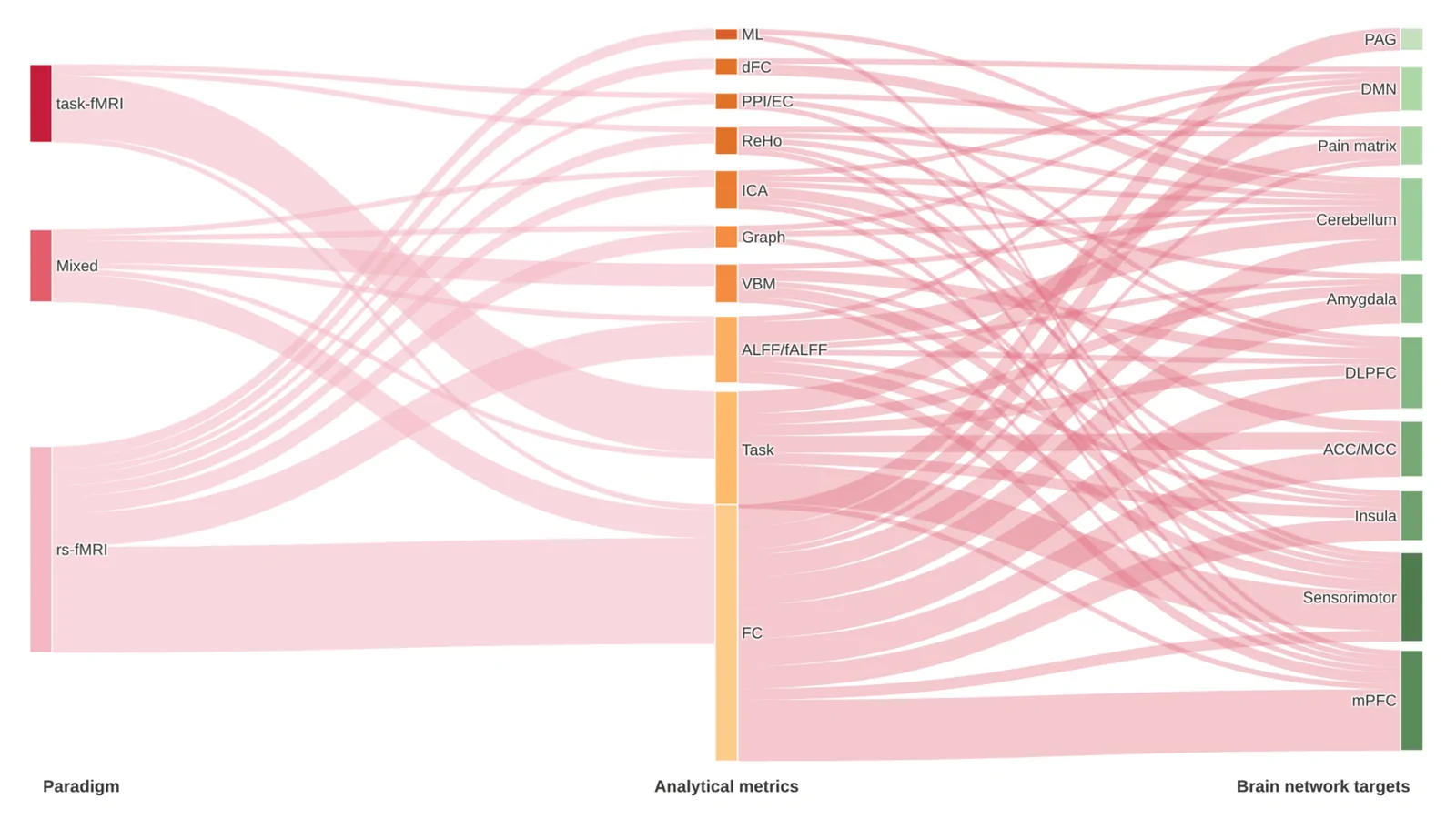
Sankey diagram of co-occurrence among imaging paradigms, analytical metrics, and brain network targets (top-10 analytical metrics and brain network targets)

Discussion

Principal Findings

This updated bibliometric analysis characterized 43 original brain fMRI studies of knee osteoarthritis (KOA) published between 2008 and 2027. In contrast to the broad-spectrum knee MRI bibliometric study by Chen et al. [22], which was dominated by structural imaging trends, the present analysis isolates functional neuroimaging, and three patterns are salient. First, resting-state fMRI was the most frequently used paradigm (24 studies, 55.8%) and accounted for 16 of the 19 studies published between 2022 and 2026, whereas task-based designs concentrated in the earlier years of the study window. Second, analytical practice converged on functional connectivity (25 studies, 58.1%), and the most frequently investigated targets were the medial prefrontal cortex (13 studies, 30.2%) and the sensorimotor cortex (12 studies, 27.9%); network-level constructs were reported less often, with the default mode network coded in only four studies. Third, publication output was concentrated in the United States (n = 20) and China (n = 17), and institutional clustering was observed in North American, Spanish, and Australian groups.

Interpretation in the Context of the Primary Literature

The predominance of resting-state designs is plausibly pragmatic. Task-based paradigms require active participation in cognitive or motor tasks, which can be impractical for elderly KOA populations with limited mobility and variable pain levels, whereas resting-state acquisition requires only that patients lie still for several minutes. At the same time, the field's convergence on functional connectivity means that between-study comparability depends heavily on preprocessing and analytical choices, which are known to alter neuroimaging results [70]; robust preprocessing pipelines exist [71-72], but the heterogeneity of imaging parameters, analysis techniques, and network definitions observed in this dataset limits direct comparability [70,72]. These observations describe methodological preference, not superiority: the frequency with which a method is used does not establish its accuracy or reproducibility.

The target distribution is consistent with the mechanisms emphasized in the primary literature. Structural imaging of the knee often fails to explain clinical variability, particularly persistent pain following technically successful total knee arthroplasty [1-2,73-75], and central sensitization has been proposed as a mechanism linking cognitive and affective components to KOA pain [8,11-12]. The frequent investigation of the medial prefrontal cortex, sensorimotor cortex, insula, and cingulate cortex in this dataset aligns with fMRI reports of altered connectivity in these regions among patients with chronic knee pain [15-17] and with meta-analytic evidence of disrupted default mode connectivity and altered top-down modulation across chronic musculoskeletal pain [16-18]. Notably, although the default mode network is a prominent framework in chronic pain neuroscience [76], it was coded as a target in only four studies in this dataset, indicating that KOA fMRI research more often reports regional than network-level targets. Preoperative central sensitization has been linked to persistent postoperative pain [13-14]; whether imaging-derived measures can contribute to such prognostic questions is a hypothesis that can only be tested in prospective clinical studies and cannot be inferred from publication patterns.

Interpretation of the "hip" Keyword

The keyword "hip" appeared exclusively in the Keywords Plus field and not among author keywords. Because Keywords Plus terms are derived from the titles of cited references, this pattern indicates that KOA fMRI studies cite hip osteoarthritis literature, particularly arthroplasty outcome and pain-mechanism studies, without designating hip OA as an author-level topic. As a by-product of citation indexing rather than author-designated content, "hip" cannot be interpreted as an emerging research frontier or a conceptual transition within this field; it is reported here only as a descriptive cross-citation phenomenon.

Limitations and Future Directions

Several limitations must be acknowledged. First, the high proportion of excluded records (56 of 99 screened) reflects an inherent limitation of database indexing: no dedicated MeSH term or field tag distinguishes brain neuroimaging from local knee MRI in the KOA literature, necessitating broad initial retrieval followed by manual screening. This approach maximizes sensitivity, and eligibility was verified through dual independent screening with chance-adjusted agreement; nevertheless, some subjectivity in classification cannot be excluded. Second, the included dataset is small (n = 43), with annual counts ranging from one to six. All temporal patterns reported herein are therefore descriptive observations rather than inferential trends, and their generalizability is limited [22]; accordingly, no annual growth rates or statistical trend tests were calculated. Third, restricting the search to the Web of Science Core Collection (SCI-EXPANDED and SSCI) and English-language publications may have introduced database and language biases, potentially omitting relevant studies from regional journals or non-English literature [24,69,77]; future updates should incorporate Scopus, PubMed, and regional databases to capture non-English and grey literature. Fourth, despite the increasing adoption of standardized methods, considerable heterogeneity remains in imaging parameters, analysis techniques, and network definitions, which limits the comparability of results [70,72]. Fifth, although the search was updated to September 6, 2026, after complete indexing of the 2025 publication year, the 2026 count represents a partial year, and one record published online in 2026 was assigned to a 2027 volume; annual counts for 2026 should be interpreted accordingly.

Sixth, consistent with its bibliometric design, this study did not include a formal quality assessment or risk-of-bias evaluation of the included publications. Bibliometric analysis examines publication metadata-authorship, affiliations, keywords, and citation networks-to characterize what has been published and how; it does not speak to the internal validity, risk of bias, or clinical robustness of the underlying primary studies. Publication frequency must therefore not be conflated with biomarker validity, thematic prominence with established clinical indication, or methodological consolidation with technical superiority or diagnostic reliability.

Future research questions that follow from these descriptive patterns include whether harmonized acquisition and reporting practices would improve cross-study comparability, and whether imaging-derived connectivity measures retain any prognostic value when tested prospectively alongside clinical and psychosocial data [74,78] and structural MRI-based frameworks [79]. Such questions can only be addressed by primary clinical studies; the present analysis maps publication activity and cannot determine clinical directions.

## Conclusions

This bibliometric analysis characterized the research landscape of brain functional MRI in knee osteoarthritis based on 43 original studies spanning 2008 to 2027 (search date: September 6, 2026). Annual publication counts fluctuated between one and six per year, peaking at six in 2022. Resting-state fMRI was the most frequently used paradigm (24 studies, 55.8%) and accounted for 16 of the 19 studies published between 2022 and 2026, whereas task-based fMRI was concentrated in the earlier years of the dataset. Functional connectivity was the most frequently applied analytical metric (25 studies, 58.1%), and the medial prefrontal cortex (13 studies, 30.2%) and the sensorimotor cortex (12 studies, 27.9%) were the most frequently investigated brain targets. Collaboration was concentrated in a United States-China dyad at the country level and in North American, Spanish, and Australian institutional clusters. Author-keyword and Keywords Plus analyses yielded complementary thematic structures comprising 10 modularity-based clusters (modularity Q = 0.6268, weighted mean silhouette = 0.8522), and the most recent keyword bursts (2024 onward) were "pain," "functional connectivity," and the Keywords Plus-derived term "hip."

These findings describe publication activity and methodological preferences within the WoSCC-indexed, English-language literature; the 2026 count represents a partial year. The reported patterns reflect what has been published and how; they do not establish the clinical validity, accuracy, or reproducibility of any imaging measure.

## Appendices

**Table 3:** Keyword harmonization thesaurus (author keywords)

Standardized term	Raw author-keyword variants merged (WoSCC export)	Total n
knee osteoarthritis	Knee osteoarthritis (n=14); knee osteoarthritis (n=7); Knee osteoarthritis (KOA) (n=1); knee osteoarthritis pain (n=1); knee osteoarthritia (n=1); Chronic knee osteoarthritis (n=1); Osteoarthritis, knee (n=1)	26
fMRI	fMRI (n=7); functional magnetic resonance imaging (n=5); Functional magnetic resonance imaging (n=3); Functional MRI (n=2); functional MRI (n=1); Functional Magnetic Resonance Imaging (fMRI) (n=1)	19
functional connectivity	functional connectivity (n=3); Functional connectivity (n=2); resting-state functional connectivity (n=2); functional connectivity (FC) (n=1); FC (n=1); Resting state functional connectivity (n=1)	10
acupuncture	Acupuncture (n=5); acupuncture (n=4)	9
chronic pain	Chronic pain (n=5); chronic pain (n=3)	8
osteoarthritis	osteoarthritis (n=3); Osteoarthritis (n=3); OSTEOARTHRITIS (n=1)	7
resting-state fMRI	Resting state fMRI (n=2); Resting-State fMRI (n=2); resting-state functional magnetic resonance imaging (n=2); resting-state fMRI (n=1)	7
pain	Pain (n=4); pain (n=1); PAIN (n=1)	6
magnetic resonance imaging	Magnetic resonance imaging (n=2); MAGNETIC RESONANCE IMAGING (n=1)	3
ALFF	ALFF (n=2); amplitude of low-frequency fluctuation (ALFF) (n=1)	3
fALFF	fALFF (n=1); Fractional amplitude of low frequency fluctuation (n=1); fractional amplitude of low-frequency fluctuation (n=1)	3
independent component analysis	independent component analysis (n=1); Independent component analysis (n=1); Independent component analysis (ICA) (n=1)	3
dorsolateral prefrontal cortex	Dorsolateral prefrontal cortex (DLPFC) (n=2); DLPFC (n=1)	3
insular cortex	Insular cortex (n=2); insular cortex (n=1)	3
brain activity	Brain activity (n=2); brain activity (n=1)	3
pain sensitization	Pain sensitization (n=2); pain sensitization (n=1)	3
regional homogeneity	regional homogeneity (n=1); Regional homogeneity (n=1)	2
brain	Brain (n=1); brain (n=1)	2
neuroimaging	neuroimaging (n=1); Neuroimaging (n=1)	2
moxibustion	moxibustion (n=1); Moxibustion (n=1)	2
expectation	Expectancy (n=1); Expectation (n=1)	2
exercise	Exercise (n=1); Physical exercise (n=1)	2
knee arthroplasty	Knee arthroplasty (n=1); total knee arthoplasty (n=1)	2
elderly patients	elderly patients (n=1); older patients (n=1)	2
